# Core and penumbra estimation using deep learning-based AIF in association with clinical measures in computed tomography perfusion (CTP)

**DOI:** 10.1186/s13244-023-01472-z

**Published:** 2023-09-29

**Authors:** Sukhdeep Singh Bal, Fan-pei Gloria Yang, Nai-Fang Chi, Jiu Haw Yin, Tao-Jung Wang, Giia Sheun Peng, Ke Chen, Ching-Chi Hsu, Chang-I Chen

**Affiliations:** 1 Department of Mathematical Sciences, University of Liverpool, Liverpool, Merseyside UK; 2 Center for Cognition and Mind Sciences, National Tsing Hua University, Hsinchu, Taiwan; 3 Department of Foreign Languages and Literature, National Tsing Hua University, Hsinchu, Taiwan; 4 Department of Radiology, Graduate School of Dentistry, Osaka University, Suita, Japan; 5 Neurological Institute, Taipei Veterans General Hospital, Taipei, Taiwan; 6 Department of Neurology, Tri-Service General Hospital, National Defense Medical Center, Taipei, Taiwan; 7 Department of Computer Science, National Yang Ming Chiao Tung University, Hsinchu, Taiwan; 8 Division of Neurology, Department of Internal Medicine, Taipei Veterans General Hospital, Hsinchu Branch, Hsinchu County, Taipei, Taiwan; 9 Board of Directors, Wizcare Medical Corporation Aggregate, Taichung, Taiwan; 10 Department of Medical Management, Taipei Medical University, Taipei, Taiwan

**Keywords:** Arterial input function, Ischemic stroke, Core, Penumbra, Perfusion parameters

## Abstract

**Objectives:**

To investigate whether utilizing a convolutional neural network (CNN)-based arterial input function (AIF) improves the volumetric estimation of core and penumbra in association with clinical measures in stroke patients.

**Methods:**

The study included 160 acute ischemic stroke patients (male = 87, female = 73, median age = 73 years) with approval from the institutional review board. The patients had undergone CTP imaging, NIHSS and ASPECTS grading. convolutional neural network (CNN) model was trained to fit a raw AIF curve to a gamma variate function. CNN AIF was utilized to estimate the core and penumbra volumes which were further validated with clinical scores.

**Results:**

Penumbra estimated by CNN AIF correlated positively with the NIHSS score (*r *= 0.69; *p *< 0.001) and negatively with the ASPECTS (*r *=  − 0.43; *p *< 0.001). The CNN AIF estimated penumbra and core volume matching the patient symptoms, typically in patients with higher NIHSS (> 20) and lower ASPECT score (< 5). In group analysis, the median CBF < 20%, CBF < 30%, rCBF < 38%, Tmax > 10 s, Tmax > 10 s volumes were statistically significantly higher (*p *< .05).

**Conclusions:**

With inclusion of the CNN AIF in perfusion imaging pipeline, penumbra and core estimations are more reliable as they correlate with scores representing neurological deficits in stroke.

**Critical relevance statement:**

With CNN AIF perfusion imaging pipeline, penumbra and core estimations are more reliable as they correlate with scores representing neurological deficits in stroke.

**Graphic abstract:**

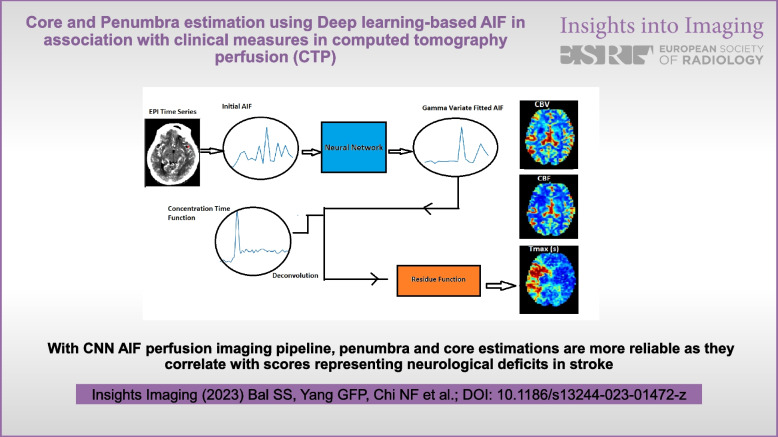

## Key points

1. CNN-based AIF improves the estimation of penumbra and infarct core volumes.
2. CNN AIF identifies patients with core regions which were ignored by conventional approaches.
3. Core and penumbra assessment with CNN AIF correlates well with clinical scores.

## Introduction

Computed tomography perfusion (CTP) analysis, which is dynamic, contrast-enhanced, and non-evasive, remains an important diagnostic tool for triaging decisions in acute stroke [1–3]. The assessment of irreversibly damaged infarct core and hypoperfused tissue or penumbra from CTP datasets is a useful paradigm for selecting stroke patients for reperfusion therapies [1, 4–7]. Following a precise assessment of the core and penumbra, cerebral reperfusion saves brain tissues that are in danger and significantly improves the patients' clinical condition [7]. Thresholding CTP parameters determine the optimal core and penumbra volumes, which are Tmax > 6 s for penumbra and relative CBF < 30% (a.u) for core [8].

Deconvolution of two time-dependent functions, concentration time function ${C}_{t}$(*t*) and arterial input function (AIF) ${C}_{a}$(*t*), is required for CTP parameter estimation (Eq. 2) [9]. The predefined contrast agent input of a large arterial vessel is referred to as the arterial input function (AIF) [10]. AIF curves, in general, have a baseline period, a first passage, and a recirculation part [11]. AIF with a distorted baseline, shape-amplitude errors in the first passage, and non-identical recirculation portions predicts incorrect perfusion parameters [12]. These inherent errors may lead to the identification of incorrect core and penumbra volumes.

The estimation of the CTP parameters can be improved by curve fitting (CF) of hemodynamic models to the AIF, according to recent studies [13]. Arterial concentration time curves plotted against time points signify that contrast agent is injected into a blood vessel upstream and dilutes downstream [11]. Curve fitting can assure an AIF with a constant baseline, a first passage with a peak amplitude, and identical recirculation portions [13, 14]. To achieve this, previous studies modeled arterial concentration time curves by using the gamma variate function, which has the value [13]:

1 $$C\left( t \right)_{{{\text{GVM}}}} = k\left( {t - {\text{AT}}} \right)^{\alpha } \exp \left( {{{ - \left( {t - {\text{AT}}} \right)} \mathord{\left/ {\vphantom {{ - \left( {t - {\text{AT}}} \right)} \beta }} \right. \kern-0pt} \beta }} \right)$$

Here *t* represents the time points, AT is the bolus arrival time, *K* is the constant scale factor, and to describe the shape of the curve *α* and *β* are used as arbitrary parameters.

Due to their ability to accurately predict AIF and final stroke lesions from acute brain imaging data, deep convolutional neural networks (CNNs) methods have been significantly used in CTP imaging in recent years [2, 15, 16]. The benefit of CNNs is that they exhibit human-like cognition and automatically extract features from brain images using numerous hidden layers [17]. There is no requirement for manual feature identification; instead, CNN models predict AIF and core with accuracy in a short amount of time, directly assisting in patient diagnosis [2, 15, 17, 18].

Apart from the thresholded perfusion parameters, medical professionals also refer to the NIHSS and ASPECT scores to make thrombectomy decisions [19–22]. Clinical trials require a severity assessment; NIHSS is considered as the gold standard for stroke severity rating as it has been shown to be a predictor of both short- and long-term outcome of stroke patients [23, 24]. In the NIHSS scale (0–42), score of 1–4 represents minor stroke, 5–15 indicates moderate stroke, 16–20 characterizes moderate to severe stroke, and a score of 21–42 indicates that the patient has severe stroke [23].

ASPECTS measures early ischemic changes in anterior circulation hyperacute ischemic stroke [25]. For ASPECTS, a normal brain has a score of 10 and the score falls as more brain regions are affected [26]. Patients with ASPECT score 0–5 benefit from mechanical thrombectomy without increasing the risk of symptomatic intracerebral hemorrhage [27]. The American Stroke Association recently updated their stroke management guidelines, and one of the key selection criteria now includes ASPECTS. Patients with baseline ASPECTS ≥ 6 are advised to receive endovascular therapy [28].

The current study investigates whether AIF estimation based on CNN, which combines features of consistent baseline, peak amplitude, and identical recirculation portions, improves volumetric assessment of core and penumbra. It is hypothesized that core and penumbra assessment with CNN AIF could be validated with clinical scores such as NIHSS and ASPECTS. We expect CNN-based AIF to identify core and penumbra in stroke patients where traditional AIF methods fail. This may aid clinicians in making thrombectomy and reperfusion therapy decisions.

## Methods

### Patient population

The current study used CTP datasets of 160 ischemic stroke patients with large-vessel occlusions (male = 87, female = 73, median age = 73 years). These datasets were obtained from the Veterans General Hospital in Taipei and its branch in Hsinchu, Taiwan. The institutional review boards for human studies gave this study their ethical approval (IRB-TPEVGH 2021-06-016 BC, IRB-2020-02-006B).

### Imaging protocol

A dose of 70 mL of contrast agent (iodine) was injected at a rate of 4 mL per second. Images were acquired on a clinical CT scanner (Phillips: Ingenuity CT) using a sequenced acquisition (KVP/X-ray Tube Current/slice thickness/slices 80 kv/190 mA/5/16) with a 24-h onset-to-imaging time. Three experienced neuroradiologists scored the NIHSS and ASPECTS, with the median NIHSS being 10 (4–19) and the median ASPECTS being 8 (6–10).

### Core and penumbra estimation from CNN-based AIF

**Fig. 1:**
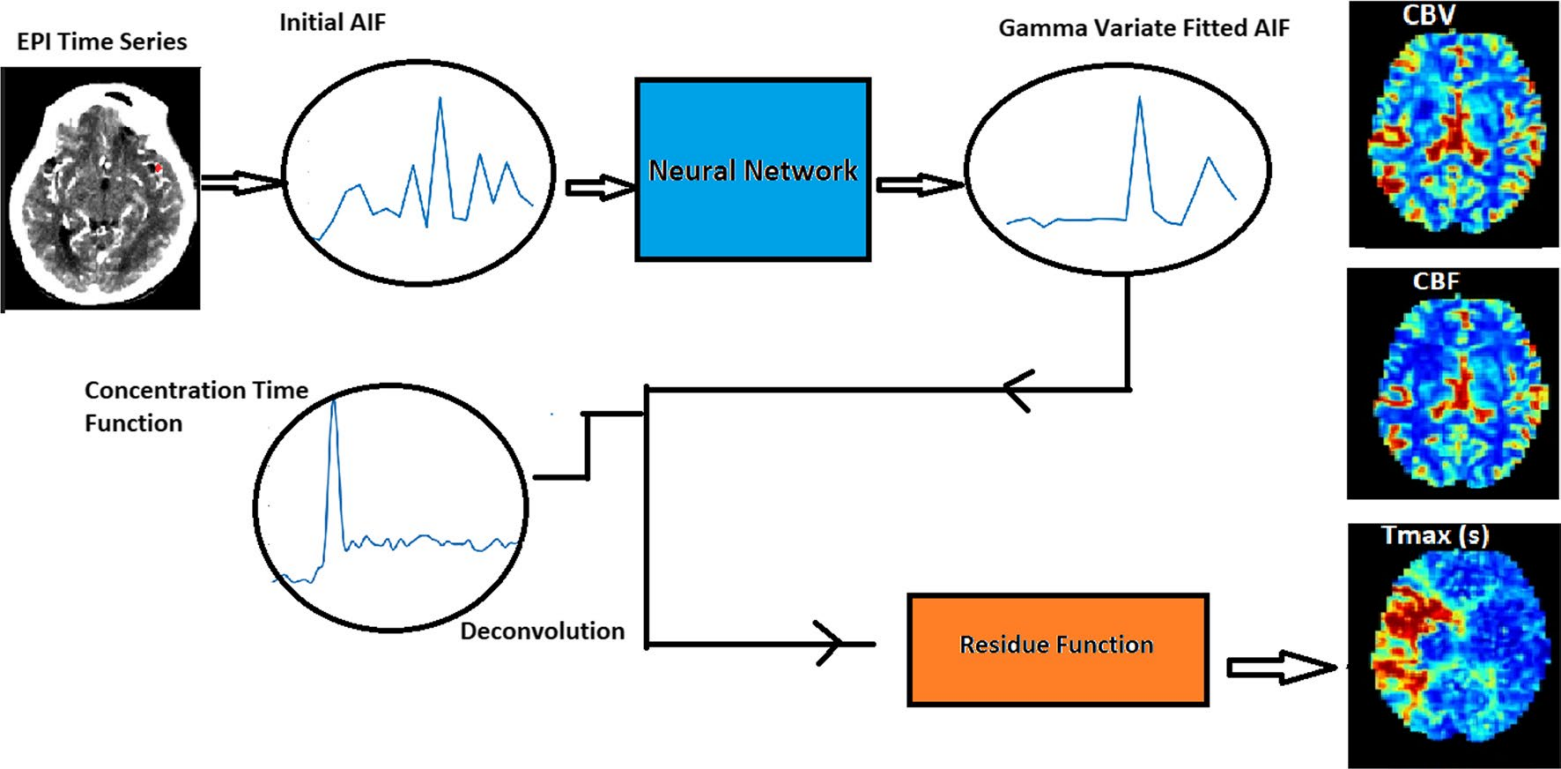
Workflow to estimate perfusion parameters

The workflow to estimate perfusion parameters is demonstrated in Fig. 1. Initially, CTP datasets were randomly divided into training (128 datasets) and validation datasets (22 datasets). A clustering algorithm is used to segment AIF labels for the CNN [29]. The algorithm draws a region of interest (ROI) in the MCA region. Then, it uses a repeated clustering analysis on the ROI to segment the AIF voxels. For more details of the method and codes used, readers can refer to [29]. Clustering algorithm estimated three AIFs for each dataset in the training and validation sets to generate training and validation AIF curves. To estimate the AIF, this clustering algorithm employs recursive cluster analysis in the middle cerebral artery (MCA) region [29, 30]. Training dataset density was improved for model training performance using feature augmentation and spline interpolation. Mirroring, rotation, and both mirroring and rotation were used for data augmentation. Initially, AIFs obtained from the clustering algorithm on 128 training datasets contain intrinsic errors associated with shallow peak, distorted baseline, and non-identical recirculation phase.

Training AIF curves are curve fitted (CF) to gamma variate hemodynamic function (C_GVM) (Eq. 1) by well-known past algorithms in MATLAB and used as labels to adjust these shape-based errors [14]. Following augmentation, 1152 distorted arterial curves estimated by the clustering method were used as input sources, and 1152 curve fitted arterial curves were used as labeled data for the CNN's supervised training.

The CNN model architecture was built with Python's Keras library. The CNN architecture consists of a single input layer that accepts input in the form of an interpolated AIF curve (500 points), two convolutional layers (kernel length = 2), a pooling layer, a flattening layer, and two dense layers that are fully connected to the output layer. To connect the convolutional layer with the average pooling layers, the ReLu activation function was used. To connect the 36-neuron dense layer to the output nodes, the softmax function was used. With a batch size of 32 and 17,238 total trainable parameters, the network was trained for 300 epochs. The optimizer was root-mean-square propagation with an initial learning rate of 0.001. The CNN model training was done on a workstation with hardware: Intel I5/Ram:16 GB/GPU: 1070 (8 GB).

Validation was performed on 22 datasets following model training. For the testing phase, distorted AIFs were chosen from a location other than the AIF location of the training dataset. The CNN model takes as input a distorted AIF curve and outputs a probability map of gamma variate fitted AIF curve (Fig. 1). This gamma variate fitted AIF curve obtained from trained CNN is referred as CNN AIF. Deconvolution of concentration time curves (${C}_{\mathrm{t}}$) with the CNN AIF (${C}_{\mathrm{a}}$)] estimates the residue function *R*(*t*) (Eq. 2), which further estimates core by thresholding CBF < 30% (Eq. 3) and penumbra by thresholding Tmax > 6 s (Eq. 4). The core and penumbra volumes estimated by using CNN AIF are compared with the volumes obtained from the AIF selected by the clustering algorithm.

2 $$C\left( t \right)= C_{{\text{a}}} \left( t \right) \otimes R\left( t \right)$$

3 $${\text{CBF}} = 100.60\;{\text{Max}}\left( {R\left( t \right)} \right)$$

4 $$T_{{\max }} = \arg \mathop {\max }\limits_{t} \left[ {R\left( t \right)} \right].\left[ s \right]$$

### Statistical analysis

Spearman's correlation coefficient was used to assess the agreement of the NIHSS and ASPECTS with core and penumbra volume. Mean volumetric differences were demonstrated using Bland–Altman plots. Wilcoxon signed rank test was used at the group level to compare perfusion parameters. MedCalc software (https://www.medcalc.org/) was used for all statistical analysis.

## Results

**Fig. 2:**
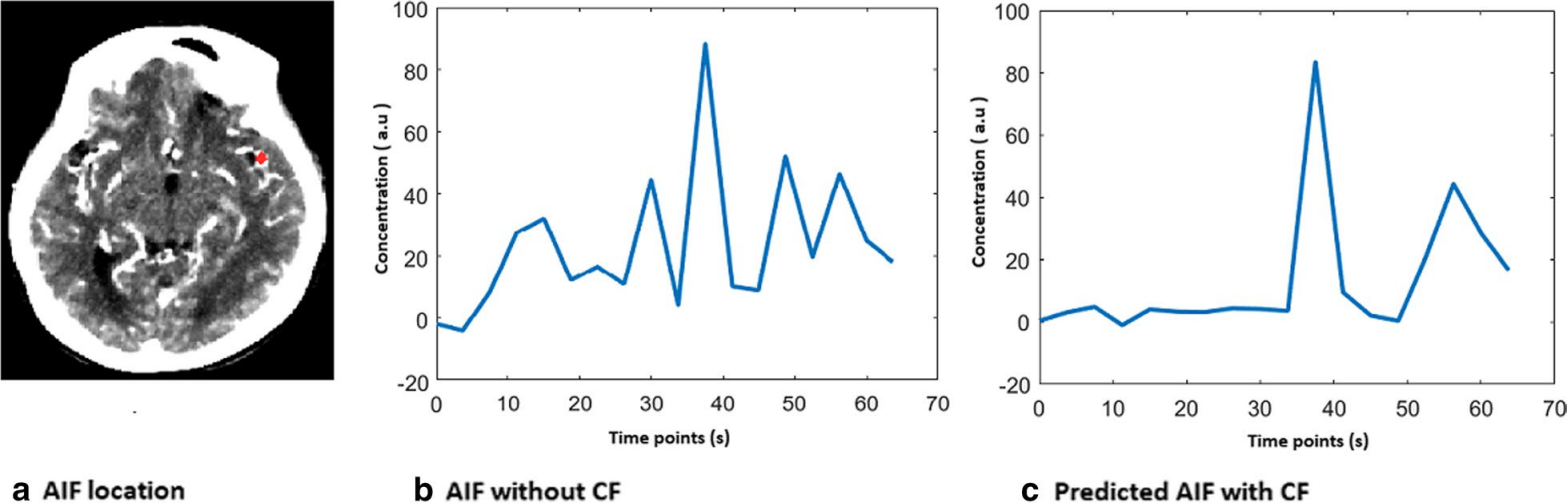
Curve fitted AIF for a single dataset (**a**) AIF location is marked as a red dot. **b** AIF curve without CF. **c** Predicted gamma curve fitted AIF curve by CNN model as an output

Figure 2 depicts the curve fitting performed by the CNN model. AIF location is marked as a red dot on an axial CTP slice for a single dataset (Fig. 2a). The raw AIF is the concentration curve of this arterial location plotted against time (Fig. 2b). The CNN model accepts the curve as input after interpolation. As an output, the CNN model predicts gamma curve fitted AIF (Fig. 2c).

### Volumetric correlation of penumbra and infarct core with NIHSS and ASPECTS

**Fig. 3:**
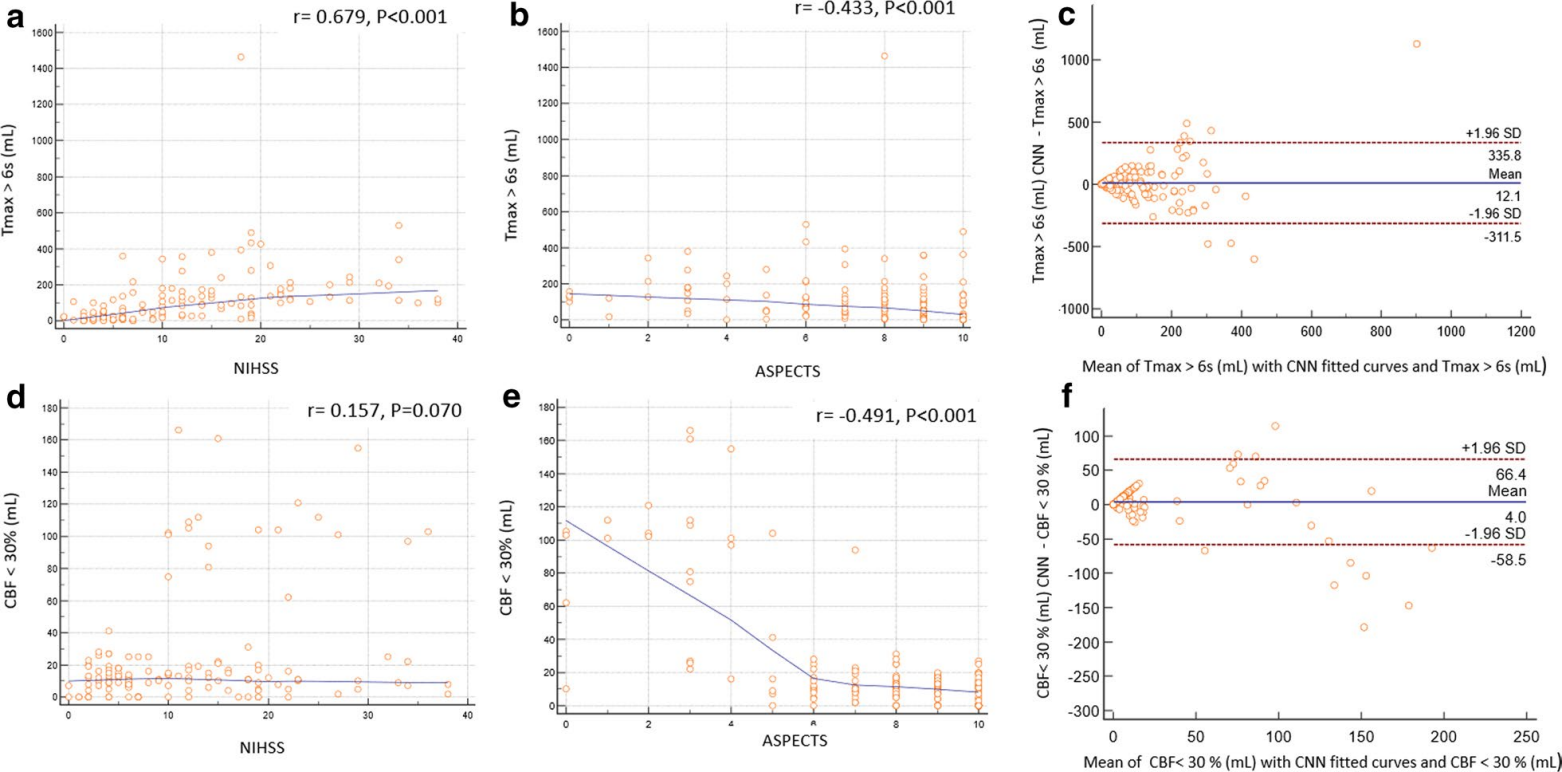
Volumetric agreement of the penumbra (Tmax > 6 s) and infract volume (CBF < 30%) with NIHSS and ASPECTS. **a** Association of Tmax > 6 s volume with NIHSS. **b** Association of Tmax > 6 s volume with ASPECTS. **c** Bland–Altman plot between the penumbra estimated by CNN CF and without CF. **d** Association of CBF < 30% volume with NIHSS. **e** Association of CBF < 30% volume with ASPECTS. **f** Bland–Altman plot between the infract core estimated by CNN CF and without CF

Tissue at risk volumes calculated with the CNN AIF was positively related to the NIHSS score (*r *= 0.69; *p* 0.001) and negatively related to the ASPECTS (*r *=  − 0.43; *p* 0.001). (Fig. 3a, b). Table 1 shows that when penumbra volume is estimated using the CNN AIF, it has a stronger positive correlation with the NIHSS score. When Tmax > 4 s, 8 s, and 10 s volumes are calculated using the CNN AIF, they have a higher positive NIHSS correlation (Table 1). According to the Bland–Altman plots, the mean volumetric difference between the tissue at risk estimated by the CNN AIF and without CNN AIF was 12.1 mL (limits of agreement, − 311.5 to 335.8 mL; Fig. 3c).

**Table 1:** Association between CT perfusion parameters and NIHSS scores (Spearman’s correlation)

	CNN CF		Without CF	
	*r*	*p* value	*r*	*p* value
Tmax > 10 s	0.70	< 0.001	0.50	< 0.001
Tmax > 8 s	0.71	< 0.001	0.54	< 0.001
Tmax > 6 s	0.68	< 0.001	0.54	< 0.001
Tmax > 4 s	0.58	< 0.001	0.46	< 0.001

Infarct core estimated by CNN AIF correlated negatively with the ASPECTS (*r *=  − 0.49; *p *< 0.001) (Fig. 3e). Table 2 shows that Tmax > 6 s volumes estimated from CNN AIF have higher negative correlation with the ASPECT score. Infarct core prediction using CNN AIF or without it both demonstrates negative and similar correlation to ASPECTS (Table 2). Mean volumetric difference for the infarct region estimated from CNN AIF without CNN AIF was 4.0 mL (limits of agreement, − 58.5 to 66.4 Ml; Fig. 3f).

**Table 2:** Association between CT perfusion parameters and ASPECTS scores (Spearman’s correlation)

	CNN CF		Without CF	
	*r*	*p* value	*r*	*p* value
Tmax > 8 s	− 0.40	< 0.001	− 0.45	< 0.001
Tmax > 6 s	− 0.43	< 0.001	− 0.39	< 0.001
Tmax > 4 s	− 0.35	< 0.001	− 0.25	0.003
CBF < 20%	− 0.46	< 0.001	− 0.47	< 0.001
CBF < 30%	− 0.49	< 0.001	− 0.49	< 0.001

### Group-level analysis

Group comparison of penumbra and core volume for stroke patients with CNN AIF and without CNN AIF is demonstrated in Table 3. Wilcoxon signed rank test indicated that the median CBF < 20%, CBF < 30%, CBF < 38%, Tmax > 10 s, volumes estimated with CNN AIF were statistically significantly higher. The median infarct core (CBF < 30%) with CNN AIF is 12 mL, whereas without CNN AIF the median core volume is 0 (Table 3). As these people have stroke, so the median of infarct core volume as zero might not reflect the hypoperfusion in patients.

**Table 3:** Mean and median volume comparison of CT perfusion parameters (Wilcoxon signed rank test)

	Mean with CNN AIF (mL)	Mean without CNN AIF (mL)	Median CNN AIF (mL)	Median without CNN AIF (mL)	*p* value
CBF < 20%	13	10	3	0	< 0.001
CBF < 30%	23	19	12	0	< 0.001
CBF < 38%	32	25	18	0	< 0.001
Tmax > 10 s	70	50	27	6	0.002
Tmax > 8 s	86	67	47	27	0.012
Tmax > 6 s	113	101	64	55	0.386
Tmax > 4 s	243	213	140	163	0.416

**Fig. 4:**
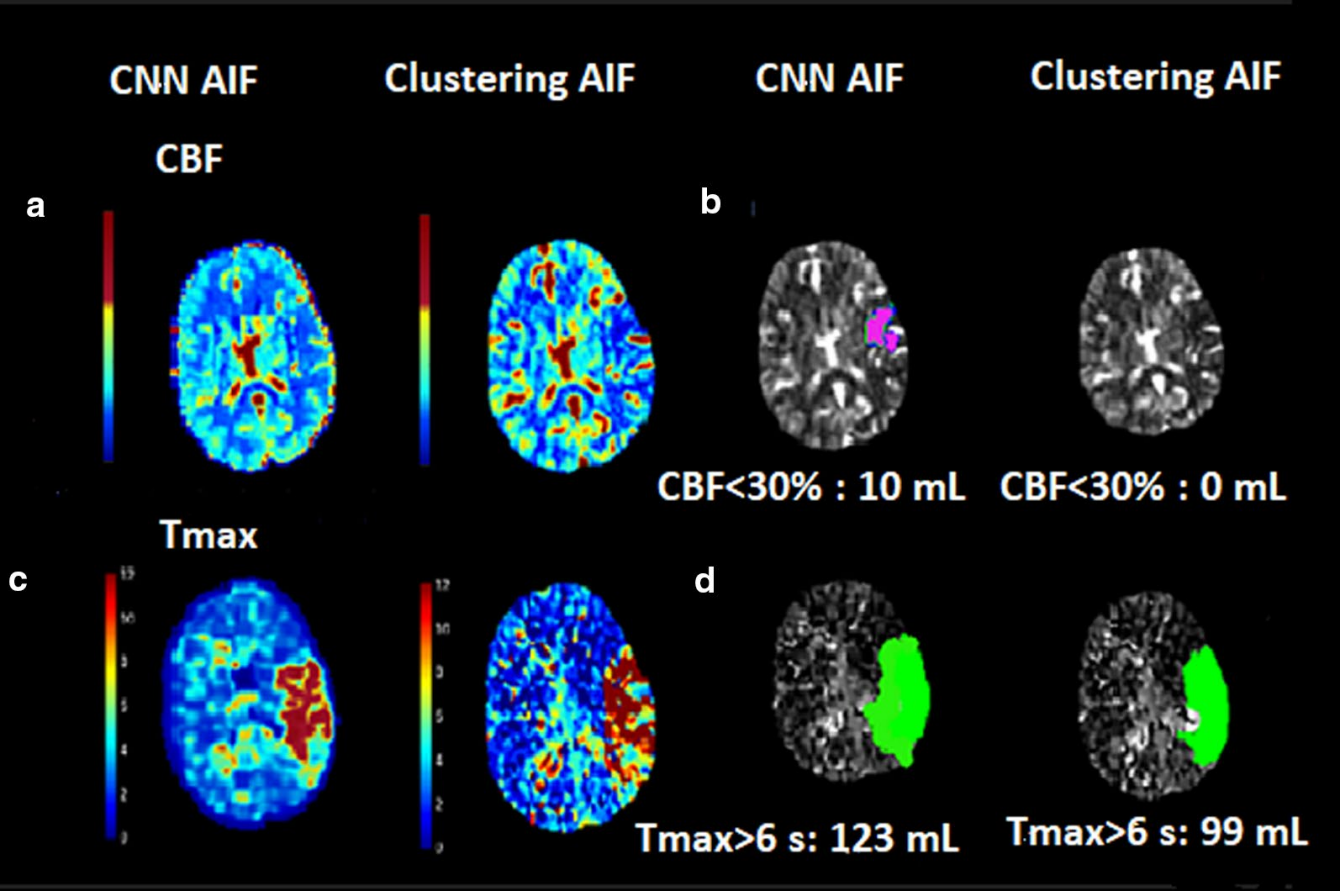
Comparison of infarct core and penumbra for a patient with severe stroke. **a** CBF maps derived with CF CNN AIF and without CF. **b** Infarct core (CBF < 30% mL) estimated with CF CNN AIF and without CF. **c** Tmax maps derived with CF and without CF. **d** Penumbra (Tmax > 6.0 s mL) estimated with CF CNN CF and without CF

In some cases, penumbra and core regions estimated without CNN AIF were not rational for patient symptoms suffering from severe stroke. These patients were typically scored with higher NIHSS (> 20) and lower ASPECTS (< 5). We demonstrate this visually with an example of a stroke patients in Fig. 4. Patient 1 was reported for NIHSS score of 23 (severe stroke), ASPECTS of 0 and acute occlusion of the left main coronary artery (LMCA occlusion). Without CNN AIF, penumbra volume was 99 mL along with absence of infarct core on left side representing flawed estimates. With CNN AIF, penumbra volume was 123 mL and volume of core region was 10 mL (Fig. 4).

**Fig. 5:**
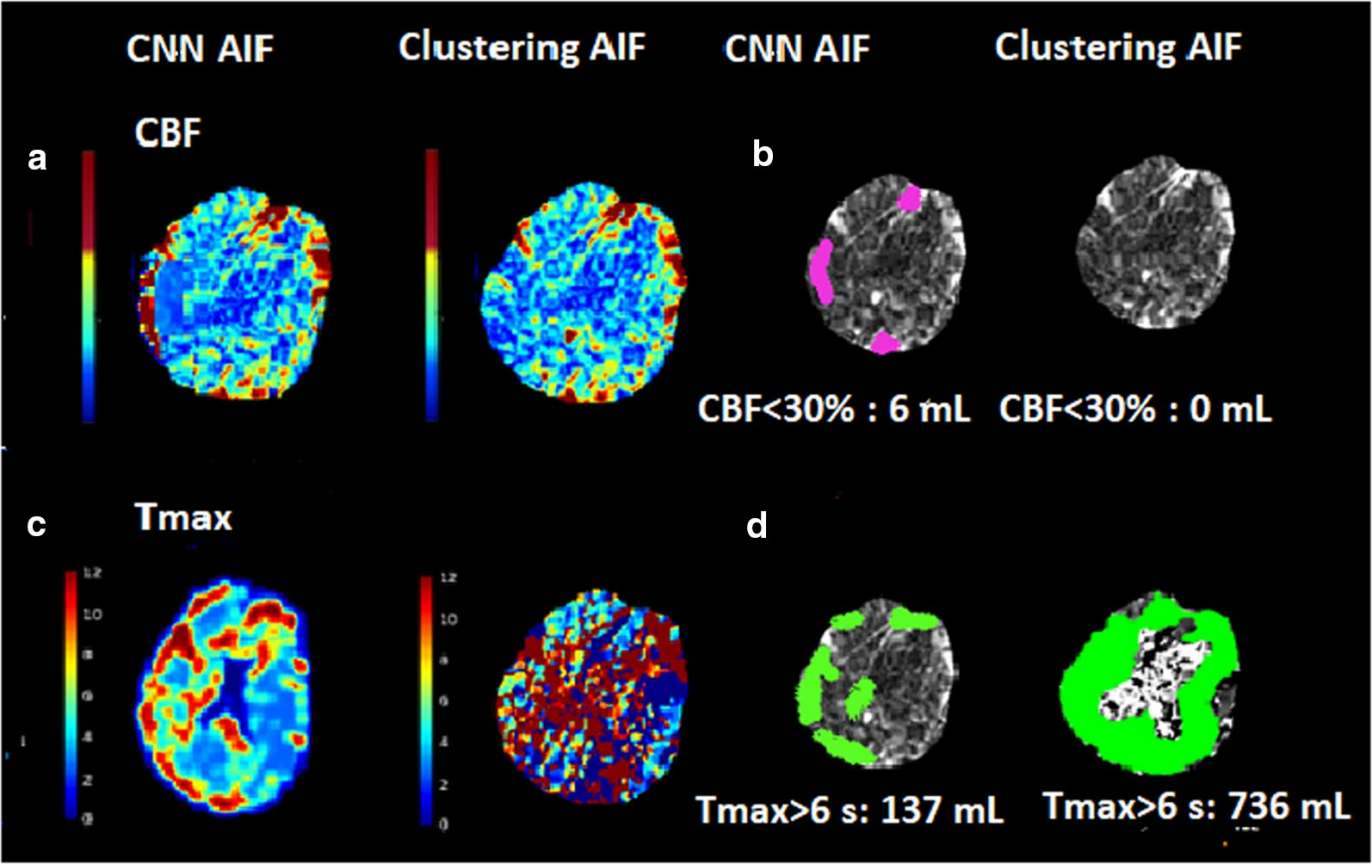
Comparison of infarct core and penumbra for another patient with severe stroke. **a** CBF maps derived with CF CNN AIF and without CF. **b** Infarct core (CBF < 30% mL) estimated with CF CNN AIF and without CF. **c** Tmax maps derived with CF and without CF. **d** Penumbra (Tmax > 6.0 s mL) estimated with CF CNN CF and without CF

Another stroke patient had a NIHSS score of 23 (severe stroke) and right internal carotid artery (RICA) occlusion. This patient reported for ischemic region at bilateral brain without CNN AIF, which was not reasonable for his symptoms as ischemia should be on the right side only. Penumbra estimated without CNN AIF was 736 mL along with absence of infarct core (Fig. 5). With CNN AIF, penumbra volume was 137 mL and volume of infarct core was 6 mL along with presence of ischemic and core region mostly on right side (Fig. 5). Out of 150 stroke patients there were 30 cases where core of patients with higher NIHSS score (severe stroke) was reported as zero without using CNN AIF. With CNN AIF mean core volume for these patients was 5 mL with median of 2 mL.

## Discussion

In this study, we study the effect of using a CNN curve fitted AIF on the perfusion parameters. CNN AIF estimates penumbra and infarct core size matching with the patient symptoms. Curve fitting approach had better correlation of penumbra and infarct volume with NIHSS and ASPECT scores, which are quantitative measures of stroke-related neurological deficits.

A recent study investigated whether the NIHSS score could be used to predict the short-term outcome of patients with intracerebral hemorrhage [31]. The NIHSS was found to have a significant positive correlation with intracerebral hematoma volume, as well as an independent predictive value of 30-day mortality [31]. A previous study found a link between NIHSS scores and the results of an arteriography performed within the first hours after an ischemic stroke. Arteriography is likely to reveal a vessel occlusion in patients with an NIHSS score of 10 [32]. Another previous study found a higher correlation (*r *= 0.96, *p* 0.001) between perfusion parameters and NIHSS by comparing PWI lesion volume to NIHSS [33]. In patients with large-vessel occlusion, a recent study discovered a stronger correlation between ASPECTS and CTP core volume [34]. These results support the use of ASPECTS and NIHSS scores as a surrogate marker for validation of penumbra and core estimation [31, 32].

Traditional AIF calculation is done either manually or by using automatic methods such as clustering and arterial likelihood methods [11, 30]. For manual AIF selection, a trained clinician operator based on his experience selects a small number of pixels on brain image as AIF [11, 29]. In arterial likelihood methods, the AIF detection algorithm uses a cost function to search for locations with signals of above-average amplitude over the entire brain slice [33]. The clustering-based method uses a recursive cluster analysis to select the AIF voxels [29]. Manual estimation of AIF is not preferred due to procedure reproducibility [29]. Flawed selection of AIF from arterial likelihood method arises from multiple penalty factors used for cost function. Conventional AIF calculation approach without fitting the AIF to a hemodynamic model could yield penumbra and core volumes having a mismatch with patient symptoms [11].

The deconvolution-based model without CF used in recent stroke studies to estimate ischemic regions is linear, despite the fact that estimation of core and penumbra depends on a variety of factors [2, 11, 35]. Certain risk factors for ischemia include distorted CTP signals, collateral status, and gray/white matter content [15, 34]. Using perfusion thresholds without correcting for distorted AIF signals may result in negligible core estimates. Fitting distorted AIF to hemodynamic models greatly reduces distortion in the recirculation and baseline parts of the AIF curve (Fig. 2) [13, 14]. The advantage of the CNN-based AIF algorithm is that it solves the AIF distortion problem and allows for the selection of the best AIF curve corresponding to distorted data points while ignoring noise or errors.

In the current study, certain stroke patients with high NIHSS and lower ASPECT scores underwent thrombectomy and reported for flawed perfusion parameter estimates. There was no core volume reported for these patients (see Figs. 4, 5). As a result, in the group analysis, the median core volume in the absence of CNN AIF was reported as zero mL (Table 3). With CNN AIF, these patients had specific penumbra and core volume. The inclusion of the CNN AIF in perfusion parameter calculations is clinically significant because estimation of core and penumbra volumes validated by scores could aid in triaging thrombectomy and reperfusion decisions.

Despite the fact that secondary prevention is started right away after utilizing information from visual assessment of core and penumbra, recurrent stroke still poses a hurdle. Recent studies discussed the potential of 3D CNN to assist accurate prognostication in recurrent stroke patients [36]. 3D deep learning-based risk prediction system (RPS) was purposed to predict the probability of stroke recurrence in form of stroke recurrence risk score (SRR score) [36]. This study concluded that deep learning-based models can outperform conventional risk scores with higher accuracy of penumbra estimation [36]. Another study proved that 3D CNN is feasible for recurrent ischemic stroke detection from computed tomography angiography source images [37]. Infracted regions were labeled with a 0.93 sensitivity and 0.82 specificity in patients who had been diagnosed with a recurrent stroke [37].

There are some limitations associated with the implementation of CNN-based algorithm. The results in the present study are based on CTP with limited dataset for training and limited choice of ground truth used for CNN model. It would be interesting to see how curve fitting of hemodynamic models other than gamma variate function would associate with NIHSS scores. The training of CF CNN model may be further improved with use of larger datasets along with use of other relevant clinical and imaging variables. It would also be interesting to apply curve fitting in perfusion analysis of magnetic resonance perfusion (MRP) datasets to see whether it characterizes core and penumbra at a similar level. Also, in spite of advancement in AI and deep learning algorithms in the field of medical imaging, the final decision regarding the patients' management and practical implementation of these algorithms will always rely on the discretion of the treating physician.

In patient cohort with acute focal neurological impairments, it is crucial to quickly identify stroke mimics, such as seizures [38]. Major use of CTP is to identify stroke patients who may benefit from reperfusion therapy [32]. Recent studies suggested CT perfusion as a promising tool for determining the cause of seizures [38]. The present study has been done on limited CTP dataset of ischemic stroke patients. The availability of CTP and MRP datasets of a different patient cohort is subject to protocols and ethics approval from the clinics. It would also be interesting to apply curve fitting in perfusion analysis of CTP datasets in first seizure patients to see whether it characterizes the seizure activity at a similar level.

In conclusion, CNN-based AIF improves the estimation of penumbra and infarct core volumes. Better correlation of penumbra and infarct volume with NIHSS and ASPECT scores was obtained using the CNN AIF. This serves as a motivation as well as evidence to include CF prior to Tmax/CBF estimations. CF AIF could identify patients with core regions likely to be ignored by conventional approaches. The inclusion of CF AIF can provide physicians with reasonably accurate and precise perfusion parameter brain images and statistics that may aid them to determine suitable triage, transport, and treatment decisions for stroke patients.

## Data Availability

The data that support the findings of this study are available from the corresponding author upon reasonable request.

## Abbreviations

AIF: Arterial input function
ASPECTS: Alberta Stroke Programme Early CT Score
CBF: Cerebral blood flow
CF: Curve fitting
CNN: Convolutional neural networks
NIHSS: National Institutes of Health Stroke Scale
